# Supplementary material for: Political and personal reactions to COVID-19 during initial weeks of social distancing in the United States
Source: PLoS One. 2020 Sep 24;15(9):e0239693. doi: 10.1371/journal.pone.0239693 (PMC7514047; doi:10.1371/journal.pone.0239693)
Supplement: Attachment — Submitted filename: Response to Reviewers.docx [file pone.0239693.s001.docx]

Response to Reviewers

Editor Comments:

| **Comment** | **Response** |
| --- | --- |
| I suggest structuring your paper according to the Strobe Guideline for cross-sectional studies | A thorough read-through of the paper has been done and small edits and additions have been made to better reflect the suggested outline given in the STROBE guidelines for cross-sectional studies. Thank you for pointing us to this resource. |
| PlosOne guidelines asks to include page numbers and line numbers in the manuscript file. | This has been done. |

Journal Requirements:

| **Comment** | **Response** |
| --- | --- |
| Please ensure that your manuscript meets PLOS ONE's style requirements, including those for file naming. | This has been done. Continuous number lines were added through the end of the manuscript, p-values were edited to no more or less than three decimal places per journal specifications, and the manuscript has been submitted in the correct format. |
| We note that you have indicated that proceeding to complete the survey was taken as implied consent in your Ethics statement. However, the manuscript indicates that informed consent was provided prior to the survey. Please correct and clarify. If implied consent please indicate whether this consent method was reviewed and approved by your Institutional Review Board. | Consent was implied with the continuance to enter the survey after reading the following: “The completion of this survey implies your consent to participate. If you choose to participate, please proceed with the questions.” The manuscript has been corrected to reflect the use of implied consent.  This method of consent was reviewed and approved by the Brigham Young University Institutional Review Board.  IRB NUMBER: IRB2020-141  IRB APPROVAL DATE: 03/25/2020 |
| Please note that PLOS ONE uses a single-blind peer review procedure. We would therefore be grateful if you could include in the information that has been redacted for peer review in the manuscript. | This has been done. |
| We note that you have stated that you will provide repository information for your data at acceptance. Should your manuscript be accepted for publication, we will hold it until you provide the relevant accession numbers or DOIs necessary to access your data. If you wish to make changes to your Data Availability statement, please describe these changes in your cover letter and we will update your Data Availability statement to reflect the information you provide. | We have published our data as well as the accompanying codebook and survey on OpenICPSR. https://doi.org/10.3886/E119629V1 |
| We note that you have included the phrase “data not shown” in your manuscript. Unfortunately, this does not meet our data sharing requirements. PLOS does not permit references to inaccessible data. We require that authors provide all relevant data within the paper, Supporting Information files, or in an acceptable, public repository. Please add a citation to support this phrase or upload the data that corresponds with these findings to a stable repository (such as Figshare or Dryad) and provide and URLs, DOIs, or accession numbers that may be used to access these data. Or, if the data are not a core part of the research being presented in your study, we ask that you remove the phrase that refers to these data. | We have included a table (Table 6) summarizing the bivariate and adjusted logistic regression analysis for general knowledge data. Edits and additions have been made to the methodology, results, and discussion sections to properly address the information presented in the table. |

Reviewer #1 Comments:

| **Comment** | **Response** |
| --- | --- |
| There are some key citations left out. Since the study also evaluated mental health outcomes such as depressive symptoms, recent literature concerning the impact of the COVID-19 pandemic on mental health should be considered. In the Introduction section, it would be good to provide a brief overview of the few available studies investigating COVID-19-related mental health outcomes. In this regard, some recent articles: [Suggested articles] | Articles discussing the mental health impacts of the COVID-19 pandemic have been referenced and discussed in the Introduction section. Thank you for your comments and recommendations. |

Reviewer #2 Comments:

| **Comment** | **Response** |
| --- | --- |
| The manuscript lacks a solid theoretical foundation and the exposition of the background is limited, the introductory section is insufficient to justify the need to carry out the investigation. | Sources have been added to the introduction section to provide a stronger foundation and rationale for conducting the study. STROBE guidelines for cross-sectional studies was also reviewed and necessary changes implemented. |
| The methodology section has not been described in detail, nor the characteristics of the sample, nor statistical aspects for the estimation of the sample size, nor are data provided on the validity and reliability of the instruments used for data collection. | We have reviewed and revised our methodology section to better align with the STROBE guidelines for cross-sectional studies.  We have added a few sentences outlining the general characteristics of our sample in the results section. A full table of the sample characteristics are provided in Table 1.  The size of the sample (1030) was determined based off other similar cross-sectional survey sample studies. Because the results were anomalous and could not have been reasonably predicted, no rigorous statistical methods were used to determine an exact size of the sample.  The majority of survey items were direct questions surrounding a novel Coronavirus. As such, there were few validated tools available to us for use in measuring attitudes, impacts, knowledge, and behaviors. However, we based many of the COVID-19 specific questions off of similar survey questions asked during the 2003 outbreak of SARS and the MERS outbreak in 2012.  Additionally, our questions regarding attitudes, knowledge etc. were evaluated using a direct question (e.g. “The U.S. government has…a) not done enough in response to COVID-19, b) done the right amount in response to COVID-19, c) has done too much in response to COVID-19). Standard psychometric tests such as Cronbach’s alpha are not appropriate for these items. Additionally, proving test-retest reliability was not possible given the rapidly evolving nature of COVID-19.  Where reliability and validity information is available, such as for the PHQ-9 and the Political Polarization in the American Public Survey, that information has been added to the manuscript. |
| The results are based on descriptive statistics, so the scope of the derived statements is limited. | We acknowledge that these results were based off a cross-sectional study done about a subject on which little was known. We also acknowledge that knowledge, best practices, attitudes, and impacts have rapidly changed since the collection of these data, and therefore, the generalizability of our results are limited to adult populations in the U.S. during the early weeks of the pandemic’s influence in the U.S. These acknowledgements have been outlined in the strengths and limitations section of the discussion section. We believe the manuscript adequately represents the limitations of the study design and collected data. Nevertheless, the rapidly evolving nature of global pandemics is an ideal situation for the use of cross-sectional and other study designs that are advantageous for early stages of knowledge. |
| Finally, the discussion and conclusions do not meet the minimum standards, one of the reasons is the poor theoretical foundation in the introduction, and this does not allow a good discussion of the data. | We have included references to the Theory of Planned Behavior in our introduction section in order to strengthen its mention in the discussion section. Materials regarding mental health have also been referenced in the introduction section to provide a better rationale for that aspect of our study. We further reviewed the discussion and conclusion section and have made the alterations necessary to match the suggestions outlined in the STROBE guidelines for cross-sectional studies as well as the author instructions for PLOS ONE articles. |
